# Patients’ knowledge and perception of anesthesia and the anesthesiologist at a tertiary hospital in Mogadishu, Somalia

**DOI:** 10.1186/s12871-025-03357-8

**Published:** 2025-09-29

**Authors:** Abdalla Mohamed Hussein, Mine Bacak, Aşır Eraslan, Sertac Cimen, Irshad Ibrahim Ali, Rahma Yusuf Haji Mohamud, Osman Abubakar Fiidow

**Affiliations:** 1 Department of Anesthesiology and Critical Care, Mogadishu Somalia- Turkiye Recep Tayyip Erdogan Training and Research Hospital, Mogadishu, Somalia; 2 Department of Urology, Turkiye Recep Tayyip Erdogan Training and Research Hospital, Mogadishu, Somalia; 3 Department of Nursing, Mogadishu Somali Turkiye Recep Tayyip Erdogan Training and Research Hospital, Mogadishu, Somalia; 4 Faculty of Medicine and Surgery, Salaam University, Mogadishu, Somalia

**Keywords:** Anesthesia knowledge, Patient perception, Anesthesiologists, General surgery, Somalia

## Abstract

**Background:**

Despite the expanding scope of modern anesthesiology, a significant public knowledge deficit persists, particularly in low-resource settings. In Somalia, where anesthesia is often delivered by non-specialist providers, this gap is likely to be pronounced. This study aimed to quantify patient knowledge and perceptions of anesthesia and anesthesiologists and to identify associated sociodemographic factors.

**Methods:**

This cross-sectional study was conducted at a single tertiary hospital in Mogadishu, recruiting 495 elective surgical patients (aged ≥ 15 years). Data were collected using structured, interviewer-administered questionnaires. The knowledge score (0–6) was analyzed using a multiple linear regression model, the assumptions of which were appropriately checked to identify significant predictors.

**Results:**

Of the 495 participants, 65.5% were aware of anesthesia, yet a profound knowledge gap was evident regarding the anesthesiologist’s broader role: 96.6% were unaware of their work outside the operating room, and 67.9% were unsure of their responsibility for postoperative care. Multiple linear regression revealed that higher educational attainment (β = 0.232, *p* = 0.002), employment (β = 0.369, *p* = 0.034), and female sex (β = 0.383, *p* = 0.037) were significant predictors of higher knowledge scores.

**Conclusion:**

This study provides the first empirical data from Somalia, quantifying a substantial deficit in patient understanding of modern anesthesiology. These findings highlight an urgent need for enhanced perioperative communication and patient education. Implementing culturally appropriate educational interventions is essential to bridge this knowledge gap, empower patients, and improve their overall surgical experience.

## Introduction

Since its early beginning in the nineteenth century, the field of anesthesia has undergone constant development, evolving from a means to enable surgery to a comprehensive medical specialty [1]. Modern anesthesiologists play a crucial role far beyond the operating room, and their expertise encompasses pain management, critical care, palliative care, and emergency response, making them essential contributors across the entire spectrum of healthcare [2–4].

Despite this expanded role, a significant and persistent gap exists between the scope of anesthesiology and the public perception. This is not a localized issue but a well-documented global phenomenon. For instance, studies conducted across Africa have revealed a stark awareness deficit. A survey in Ghana found that only 53% of patients were aware of anesthesia services [5], while in a Nigerian tertiary hospital, over half of the surgical patients (53.3%) could not identify who administered anesthesia [6]. This contrasts with data from Turkey, where a higher but still imperfect 73.6% of patients correctly identified the anesthetist as a doctor [7].

However, the most profound knowledge gap lies in understanding the role of anesthesiologists outside the operating room. This is consistently the least understood aspect of the specialty. For example, less than 15% of patients in a Ghanaian study and only 16% of learners in a South African survey identified the duties of an anesthetist in the ICU or pain clinics [3, 8].

In Somalia, these challenges are compounded by a healthcare system in which anesthesia services are often delivered by non-specialist providers, potentially widening the public knowledge gap. Although the broad problem has been quantified in other settings, no specific data exist for the Somali population. Therefore, this study aimed to investigate the specific knowledge and perceptions of anesthesia and anesthesiologists at a tertiary hospital in Mogadishu, Somalia, and to identify the sociodemographic factors that influence this understanding.

## Methods

### Study design and setting

This hospital-based cross-sectional study was conducted between September and December 2023 at the Mogadishu Somali Recep Tayyip Erdogan Training and Research Hospital, a tertiary referral center and the country’s leading teaching hospital. The study involved direct interviews with patients using a structured questionnaire during the pre-anesthesia assessment.

### Eligibility and ethical considerations

All elective surgical patients aged ≥ 15 years were considered eligible for the study. Written informed consent was obtained from all participants before their inclusion in the study. Participants who declined to participate or were emotionally, physically, or cognitively unfit to be interviewed were also excluded. Ethical approval for the study was obtained from the hospital Ethics Committee (Ref: MSTH/16078).

### Sampling and participant recruitment

The required sample size was calculated using the single-population proportion formula: n = [Z² * P(1-P)]/d², which is appropriate for a descriptive cross-sectional study aiming to estimate prevalence. The parameters were set a priori as follows: 95% confidence level (Z = 1.96), margin of error (d) of 5% (0.05), and estimated prevalence (P) of 50% (0.5). A 50% prevalence was chosen as a conservative measure to yield the maximum possible sample size, ensuring that the study would be adequately powered to estimate the prevalence with the desired precision, irrespective of the true underlying proportion.

Based on these parameters, the minimum required sample size was calculated as 384. The study was conducted over a predefined four-month period (September to December 2023). A consecutive sampling approach was employed, whereby all eligible patients presenting to the pre-anesthetic clinic during the study period were invited to participate. This systematic, time-based recruitment strategy resulted in a final sample of 495 patients, which exceeded the minimum requirement and enhanced the statistical power of the study for subsequent regression analyses.

### Data collection

A team of anesthesiology experts developed a structured questionnaire to ensure its content validity. To ensure cultural and linguistic appropriateness, the questionnaire was translated into Somali and then back-translated into English by independent bilingual experts to confirm its accuracy. The tool was then pilot tested on 20 preoperative patients (not included in the final analysis) to check for clarity and comprehension of the questionnaire. Given that a significant proportion of the study population (48.3%) had no formal education, the questionnaire was administered as a structured verbal interview in the Somali language by trained data collectors.

A knowledge score (0–6) was derived by awarding one point for each of the following six correctly identified roles of an anesthesiologist: (1) administering anesthesia, (2) monitoring vital signs during surgery, (3) managing postoperative pain, (4) waking the patient up, (5) managing patients in the ICU, and (6) performing emergency resuscitation.

### Data analysis

Data were entered and analyzed using SPSS (version 26). Descriptive statistics were used to summarize patient characteristics and response frequencies. The primary outcome was the anesthesia knowledge score (0–6).

A multiple linear regression model was employed to identify the independent predictors of this score. Although the knowledge score was an ordinal variable, it was treated as a continuous variable for this analysis. This approach is common in health research when the number of categories is sufficient (≥ 5), as it provides more easily interpretable coefficients. The validity of this approach was assessed by checking the key assumptions underlying linear regression. Specifically, the residuals of the model were examined and found to be approximately normally distributed via histograms and P-P plots. Furthermore, tests for multicollinearity (Variance Inflation Factor < 2) and homoscedasticity (visual inspection of the scatterplot of residuals) indicated that the assumptions were adequately met, supporting the use of the linear regression model.

Sociodemographic variables, including age, sex, education level, and employment status, were included as independent variables. Statistical significance was set at *p* < 0.05. Results from the regression are presented as unstandardized beta (β) coefficients with 95% confidence intervals (CI).

## Results

### Sociodemographic characteristics

A total of 495 patients participated in this study were included. The majority of the participants were male (66.3%), with a mean age of 38.79 ± 17.24 years. A significant proportion (48.3%) reported having no formal education, and 56% were unemployed. More than half of the participants (50.9%) had not undergone any previous surgical procedures (Table 1).

**Table 1:** Sociodemographic characteristics of participants

Factor		Frequency %
Gender	Male	328 (66)
	Female	167 (34)
Age	15–19	30 (6)
	20–29	158 (31)
	30–39	117 (24)
	40–49	59 (12)
	50–59	47 (10)
	60 and above	84 (17)
Educational status	Never	239 (48.3)
	Primary	106 (21.4)
	Secondary	97 (19.6)
	Graduate	53 (10.7)
Occupation	Unemployed	277 (56)
	Employed	218 (44)
Previous surgery/anesthesia	No	165 (50.9)
	Yes	159 (49.1)

### Patient knowledge and perceptions

While 65.5% of the participants had heard of anesthesia, their primary source of information was friends and relatives (48.5%). Most patients (88%) understood that anesthesia was necessary for surgery; however, their knowledge of perioperative procedures was poor. Over half (51.2%) did not know the required fasting period, and 66.7% did not know the reason for fasting, with only 3.7% correctly identifying aspiration risk.

There was significant confusion regarding the identity and role of the anesthesiologist. While 75.6% identified an “anesthesia doctor” as the provider, only 57.4% knew that an anesthesiologist was a medically qualified doctor. A profound knowledge gap was observed regarding work outside the operating room, with 96.6% unaware of this broader scope. The primary intraoperative role was perceived as simply alleviating pain (57.7%), with few recognizing their responsibility for monitoring vital signs (13.3%). Furthermore, 67.9% of the respondents were unaware of who was responsible for postoperative care and often misattributed this role to the surgeon (27.5%) (Table 2).

**Table 2:** Patients’ knowledge of anesthesia and the role of the anesthesiologist

Factor	Response	Frequency (%)
Q1: Have you heard about anesthesia?	No	171 (34.5)
	Yes	324 (65.5)
Source of knowledge about anesthesia:	Friends and Relatives	157 (48.5)
	Patient	143 (44.1)
	Media	24 (7.4)
Q2: Reason for applying to anesthesia Polyclinic	Referred	270 (83.3
	Do not know	10 (3.1))
	To explain the operation, I had	37 (11.4)
	To give information about existing illnesses	7 (2.2)
Q3: Is anesthesia applied in the same way in all patients?		
	No	289 (89.2)
	Yes	35 (7.1)
Q:4 Is anesthesia needed if surgery has to be performed?	Yes	285 (88)
	Do not know	34 (10.5)
	No	5 (1.5)
What is to be stopped before surgery?	Both solid and liquid	253 (78.1)
	Only solids	46 (14.2)
	No need to stop	13 (4)
	Only liquids	9 (2.8)
	The foods are eaten little by little	3 (0.9)
Pre-op fasting period		
	Do not know	166 (51.2)
	12 h	71 (21.9)
	6 h	62 (19.1)
	24 h	16 (4.9)
	2 h	9 (2.8)
Q5: Why preoperative fasting period is essential?	Do not know	216 (66.7)
	Nausea and vomiting	49 (15.1)
	surgery can be complicated during an operation	47 (14.5)
	Risk of Aspiration	12 (3.7)
Q6: Who applies anesthesia?	Anesthesia doctor	245 (75.6)
	Nurse	41 (12.7)
	Do not know	19 (5.9)
	Anesthesia technician	17 (5.2)
	Surgeon	2 (0.6)
Anesthesiologists are?	Medically qualified doctor	186 (57.4)
	Educated person	110 (34)
	University graduate	20 (6.2)
	Non‑medically qualified doctor	5 (1.5)
	Nurse	3 (0.9)
Where do anesthesia doctors work?	Operation theater	313 (96.6)
	Do not know	5 (1.5)
	Intensive care units	3 (0.9)
	Pain units	3 (0.9)
What is the role of an anesthesia doctor during an operation?		
	Relieving pain	187 (57.7)
	Do not know	51 (15.7)
	Waking up the patient	43 (13.3)
	Monitoring vital signs	43 (13.3)
What type of anesthesia do you know?		
	General anesthesia	248 (76.)
	Do not know	25 (7.7)
	Spinal anesthesia	8 (2.5)
	local anesthesia	8 (2.5)
	all	35 (10.8)
Who is responsible for postoperative care?	I have no idea	220 (67.9)
	The surgeon	89 (27.5)
	Anesthesia doctor	12 (3.7)
	Another doctor	3 (0.9)
Postoperative pain	It has to be tolerated	168 (51.9)
	It is an undesirable condition	132 (40.7)
	It is natural	11 (3.4)
	The drug must not be used	7 (2.2)
	It is a marker of healing	6 (1.9)
Important of anesthesia doctor		
	Very important	276 (85.2)
	moderately important	36 (11.2)
	Not much important	6 (1.9)
	Do not know	6 (1.9)

#### Predictors of anesthesia knowledge

Multiple linear regression analysis identified several significant predictors of anesthesia knowledge scores (Table 3). For each unit increase in educational level (e.g., from primary to secondary), the knowledge score increased by an average of **0.232 points** (β = 0.232, *p* = 0.002). Employed participants scored 0.369 points on average, **0.369 points higher** than unemployed participants (β = 0.369, *p* = 0.034). Female participants also demonstrated higher knowledge, scoring an average of **0.383 points more** than male participants (β = 0.383, *p* = 0.037).

**Table 3:** Multiple linear logistic regression

Covariates	β(Beta)	*P*-value	95.0% Confidence Interval for B	
			Lower Bound	Upper Bound
Age	−0.008	0.102	−0.018	0.002
Education	0.232	0.002	0.083	0.382
Occupation	0.369	0.034	0.028	0.710
Sex (female)	0.383	0.037	0.023	0.744

β = beta

## Discussion

In Somalia, where non-specialists often deliver anesthesia services, our findings from this single tertiary hospital cohort highlight a significant gap in the public understanding of anesthesia. This mirrors challenges in other low-resource African settings [5, 9]but also underscores the urgent need for localized interventions. The overwhelming perception of patients that the anesthesiologist’s role is solely to ‘alleviate pain’ during the operation, with 96.6% unaware of their work outside the theatre, is a theme that resonates strongly with the international literature. Studies from Ghana [3], Nigeria [6], and Ethiopia [10] have reported poor awareness of the specialty’s role in intensive care, pain management, and emergency resuscitation medicine.

This study suggests that educational attainment plays a crucial role in patients’ understanding of anesthesiology. Our multiple linear regression analysis showed that for every unit increase in academic level, there was a corresponding increase of 0.232 in the knowledge score. This result is consistent with previous studies that have found that education is a significant determinant of health literacy, particularly in areas that require technical understanding, such as anesthesia [7, 11]. The lack of formal education in nearly half (48.3%) of the patients in our study highlights a critical knowledge gap regarding anesthesia, underscoring the need for more targeted educational interventions.

Employment status has also emerged as a predictor of anesthesia knowledge. Employed individuals demonstrated a higher level of understanding than unemployed individuals, as similar studies have found a strong association between knowledge level and employment status [12]. This can be attributed to increased access to information and medical services that typically accompany employment, as well as improved overall health literacy.

The study also found that female participants scored higher in knowledge of anesthesiology than their male counterparts. This finding is consistent with earlier research suggesting that women generally have higher health literacy [7, 8]. However, this may also reflect specific cultural or social dynamics in the study population, where women may be more proactive in seeking health information or discussing medical procedures with healthcare providers. However, it is important to note that, as a non-randomized sample, this finding could also reflect the specific characteristics of the recruited cohort. Future studies with probability-based sampling would be valuable in confirming this sex-based trend in the broader Somali population.

Additionally, although a high percentage of participants (89.2%) recognized the need for anesthesia during surgery, important misconceptions persisted, particularly regarding postoperative care. A large number of respondents (67.9%) were unsure of who was responsible for postoperative care, with many mistakenly thinking that it was the surgeon. This finding reflects global trends, where the role of anesthesiologists in postoperative care is often overlooked, as shown in studies from Nigeria and South Africa [4, 9]. This is probably because the surgeon was the main and most visible contact with the patient during their hospital stay. The anesthesiologist’s key interactions often occur when the patient is anxious before surgery or unconscious during surgery, which leads to less recall and a weaker patient-provider relationship. This phenomenon has been reported in several international studies [10].

While 85.2% of respondents recognized the importance of the anesthesiologist’s role, many remained unaware of the broader scope of their responsibilities beyond the operating room. Most participants associated anesthesiologists with intraoperative pain management, with little understanding of their role in monitoring vital signs or managing postoperative care. This aligns with previous research suggesting that patients often overlook the anesthesiologist’s comprehensive role in perioperative medicine [11, 12]. Considering the central role of anesthesiologists in ensuring patient safety throughout surgery, these knowledge gaps highlight the need for improved patient education and communication between the two parties.

Anxiety about anesthesia also emerged as a common concern, as many patients were uncertain about the risks and procedures involved. For instance, although 88% of the participants understood that anesthesia was necessary for surgery, there was confusion about the preoperative fasting guidelines and their purpose, which increased anxiety. Studies have highlighted how fear of the unknown, worries about waking up during surgery, and postoperative complications contribute to patient stress [12].

## Conclusion

This study offers the first empirical assessment of patient knowledge regarding anesthesia in Somalia, revealing a substantial deficit in understanding the role of anesthesiologists beyond intraoperative care. While most participants recognized the necessity of anesthesia during surgery, their awareness of its broader applications–including postoperative management, intensive care, and resuscitation–was limited. These findings align with those of the existing literature on other low- and middle-income countries. Sociodemographic factors, such as higher education, employment status, and female sex, were significantly associated with greater knowledge, highlighting disparities in health literacy.

The clinical implications are clear: there is a pressing need to improve perioperative communication and patient education. We recommend integrating culturally appropriate educational interventions, such as pictorial aids or short videos in the Somali language, during pre-anesthetic consultations. From a policy perspective, these findings should inform national health education campaigns aimed at raising public awareness of anesthesia. Future research should involve multicenter studies to enhance generalizability and include interventional designs to evaluate the impact of educational tools on knowledge, satisfaction, and anxiety levels.

### Limitations

The interpretation of our findings is subject to some limitations. First, the single-center design of a tertiary referral hospital limits the generalizability of our results to other healthcare settings in Somalia, such as regional or private facilities. Second, the non-probability consecutive sampling method introduces a risk of selection bias, restricting the representativeness of our cohort. Third, the cross-sectional nature of the study establishes associations between variables but precludes any inference of causality. Finally, the use of interviewer-administered questionnaires, although necessary for our population, carries an inherent risk of social desirability and interviewer bias that cannot be eliminated despite standardization.

## Data Availability

The datasets generated and/or analyzed during the current study are available from the corresponding author upon reasonable request.

## Declarations

### Provenance and peer review

Not commissioned, externally peer-reviewed.
